# Salt-bridges in the microenvironment of stable protein structures

**DOI:** 10.6026/97320630016900

**Published:** 2020-11-30

**Authors:** Amal Kumar Bandyopadhyay, Rifat Nawaz Ul Islam, Niladri Hazra

**Affiliations:** 1 Department of Biotechnology, University of Burdwan, West Bengal, India; 2 Department of Zoology, University of Burdwan, West Bengal, India

**Keywords:** Microenvironment (ME), salt-bridge, energetics, thermostability, mutagenesis, protein-engineering

## Abstract

Salt-bridges (sb) play an important role in the folding and stability of proteins. This is deduced from the evaluation of net energy in the microenvironments (ME, residues that are 4 Å away from positive and negative partners of salt-bridge and interact with them). MEs act as a determinant of net-energy due to the intrinsic features in the sequence. The stability of extremophilic proteins is due to the presence of favorable residues at the ME without any unfavorable residues. We studied a dataset of four structures from the protein data bank (PDB) and a homology model (1HM5) to gain insights on this issue. Data shows that the presence of isolated charges and polar residues in the core of extremophilic proteins helps in the formation of stable salt-bridges with reduced desolvation. Thus, site-specific mutations with favorable residues at the ME will help to develop thermo stable proteins with strong salt bridges.

## Background

The tertiary structure of a protein is made up of contributing and compromising weak forces [1] derived from the underlying amino acid sequence [2]. However, the origin for such a principle of convergence of structures and functions derived from divergence in orthologous sequences is still unknown. When conditions are recreated in the laboratory in which a protein is functional in the environment (high-temperature, high-salt, etc.), its primary sequence forms the same functional state [3,4,5]. These intrinsic codes of protein folding embedded in the primary sequence [2], which determines tertiary structure through the interconnection and interplay of weak forces and secondary structures [6]. The evolutionary pressures of the environment are responsible for the divergence of the primary sequence's codes in the orthologous set, which causes functional convergence in the related environment in tertiary structures [7]. Salt-bridge is a specific electrostatic interaction whose importance especially in protein structure specific binary design (core vs surface, local vs long-ranged, etc.), folding, and stability has been worked out [8,9,10,11]. There are two types of salt-bridges, namely isolated-pair (ip) and network-pair (nu) [12]. Although Poisson-Boltzmann Equation (PBE) has established a method of knowing the net and component energy of the salt-bridge [12, 13], network unit method (NUM) of knowing the actual energy of nu has recently been discovered [14]. Salt-bridge's net-energy depends on three terms: desolvation (ΔΔGdslv), bridge (ΔΔGbrd) and background (ΔΔGbac) [12,13,14]. The first is always costly and the second is always contributing but the third can be costly or contributing. The energy of the first two terms is dependent on the salt-bridge residue, while the third is dependent on the rest of the protein except the two in the salt-bridge. Most of ΔΔGbac contributes by a very small number of background residues, which are identified as microenvironment (ME) of the salt-bridge [15]. ME is important to understand the structure, function, stability and evolution of a protein, which is directly related to the intrinsic sequence property [14,15,16]. Aspartate-tRNA ligase is a dual specification enzyme that binds to Mg++ and nucleotides, except the substrate [17]. The enzyme has the atomic structure that covers the mesophilic and thermophilic domains, but the halophilic representative is still absent. We have used a fully automated procedure (ADSETMEASv2.0) developed in our laboratory, as it is difficult to get the above results manually. Applications from thermophilic, halophilic, and mesophilic's X-ray and model structure's outputs extracted by the use of the program are highlighted in this work. Here, we have provided evidence that the abundance of charge and polar residues has certainly played a major role in salt-bridge's stability and in extremophilic adaptation. We further show ME is composed of both favorable and unfavorable residues of which the former dominates in extremophiles. It is possible to improve the structure and function of protein by mutation of target residue from unfavorable population of ME. The above studies are also possible with homology-modeled structures. These details will not only help to innovate intrinsic factors involved in protein adaptation but also shed light on the importance of these at the residue level by protein engineering. We believe our results are the first step in finding and applying interconnections between protein sequences and structures.

## Materials and Methods:

### Sequence and structure retrieval and processing:

The sequences of aspartate-tRNA (Asp/Asn) ligase of the five species (Homo sapiens, Haloarchaea: Haloarcula marismortui, Euryarchaeota: Thermococcus kodakarensis, Thermobacteria: Thermus thermophilus, crenarchaeota: Sulfurisphaera tokodaii) were brought from the Uniprot database along with relevant annotations. Their structures were also procured from RCSB, PDB database (Table 1 - see PDF). In order to establish phylogenetic tree and determine the average properties of the sequence, homologous sequences of each species were brought from NCBI database. Sequence analysis was done through PHYSICO, PHYSICO2 [18,19]. Each structure was minimized by AUTOMINv1.0 [20] in a multi-chain format in the presence of shell water and then, only one chain is processed.

### Homology modeling and evaluation:

It was necessary to see if the homology model had a salt-bridge restoration, and if it could be used to understand the extremophilic properties. Since there is no halophilic structure in the database, we developed and evaluated it following the previous procedure [20,^21^-^26^]. Since the model quality depends on the alignment method and the loop-length, we did the alignment in T-coffee method.

### Salt-bridge model and energetic computation:

The net-energy (ΔΔG_net_) of the salt-bridge in the computation method depends on three component terms namely ΔΔG_dslv_, ΔΔG_brd_ and ΔΔG_bac_ i.e.

ΔΔG_net_ = ΔΔG_dslv_ + ΔΔG_brd_ + ΔΔG_bac_

The folded and unfolded states of the protein are considered to compute ΔΔG_dslv_, but only the first state is used for the other two [27-30]. The desolvation of the positive and negative charges of the salt-bridge is independent, so they are computed separately. In this case (unfolded state), (i+1) and (i-1) residues are included with the charge residue (i). This computation is done in both solvent and vacuum conditions for each state and for each charge. The rest of the details are discussed in detail in our and others previous publications [12,26-32]. In short, to compute component terms, atomic charge is computed by PDB2PQR [33]. All mutated states only have side-chain charges. Atomic potential file obtained by APBS [34] by solving the PBE. Atomic energy is obtained by multiplying atomic charge and conversion factor with the atomic potential. It is important to keep in mind that the PBE method (APBS) is an approximate approach and thus, it has many limitations. Proper use of these parameters i.e. Protein's size-specific grid-spacing, grid-center, Debye-Huckel boundary conditions, ionic strength, protein and solvent dielectric, surface calculation method, etc. is essential for the solution of this multi-parameter PBE method, especially in the case of proteins. The energy terms for the ip and nu salt-bridge are calculated using the IPM and NUM [14] methods respectively. In addition to residue specific salt-bridge energy, its accessibility, and bond-multiplicity, as well as mean distances are extracted by the use of NACCESS [35], and SBION and SBION2 [8,9] respectively. All of the above steps can be accomplished easily through ADSETMEASv2.0 (to be communicated). As the outputs are systematically arranged in excel, it is possible to analyze and apply them in post-run scenario.

### ME and binary details:

ΔΔGbac is the sum of the energy of all the other protein residues except the two salt-bridge residues. Nayek et al. (2015) is the first to conceptualize ME [15]. ME's main-chain atoms are excluded and converted to residue specific energy by adding side-chain atom's energy. In this case also separate methods are used for accurate determination of ME energy (ipenv, nuenv) for ip and nu type of salt-bridges. Accessibility is extracted with the help of NACCESS to know the location of these residues in core and surface of protein [35]. ME's secondary structure information (helix or sheet or coil) is calculated by PROPAB's principle [36]. Other binary data such as residue class, physicochemical properties, ME-residue's distance from positive and negative partners of salt-bridge are also extracted. These ME properties were then correlated with the properties of the sequences extracted from the protein structure and UniProt database.

## Results:

### Sequence divergence and salt-bridge's energetics

**Figure 1:**
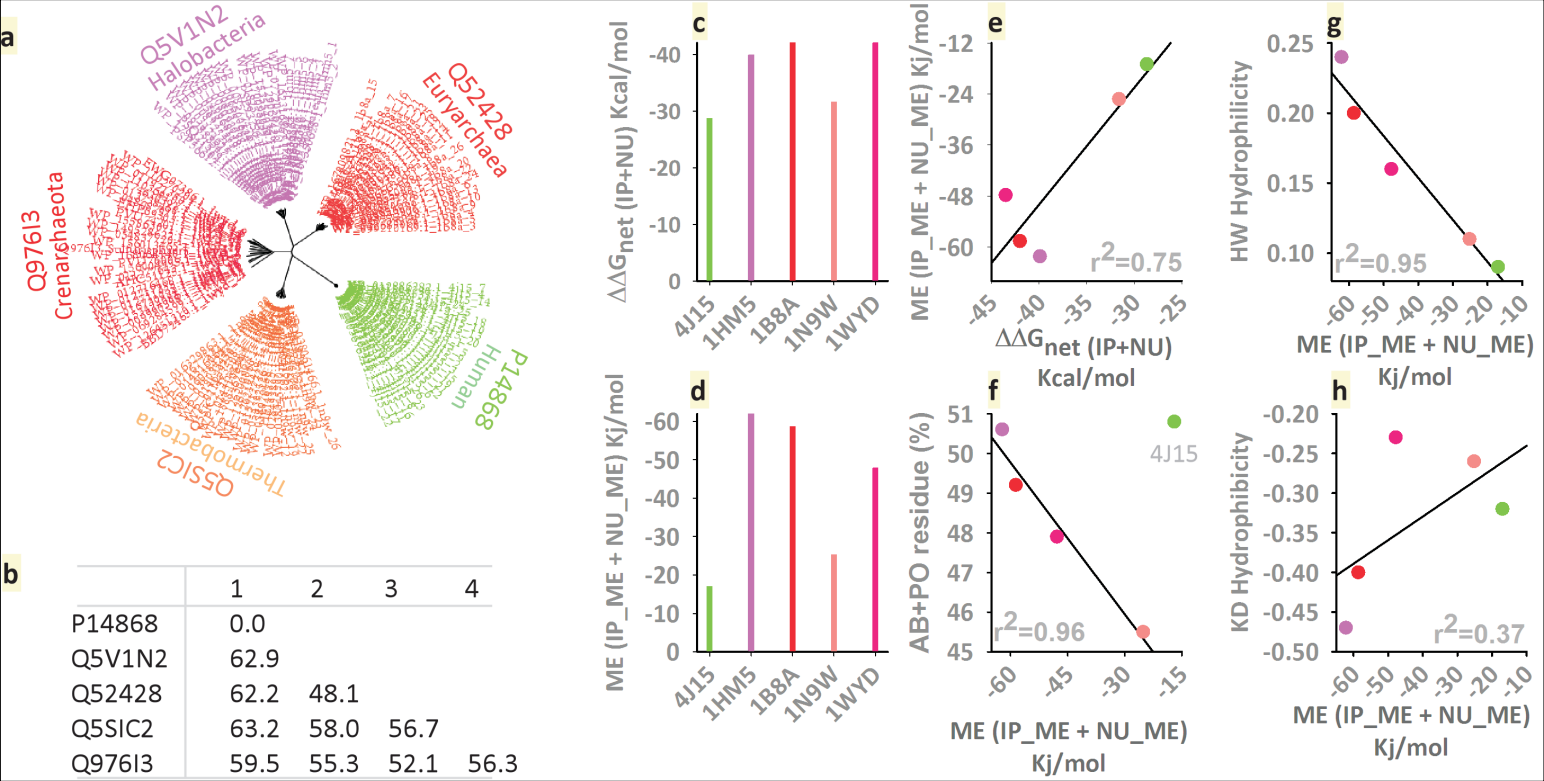
Characteristics of the aspartate-tRNA ligase sequence and the salt-bridge and their interrelationships. a) Phylogenetic relations between human (green), Haloarcula marismortui (purple), Thermococcus kodakarensis (red), Thermus thermophilus (light-red) and Sulfurisphaera tokodaii (red). Comparison of difference matrix (b), net salt-bridge energy (c) and ME energy (d) features of these microbes. Correlation between ΔΔGnet vs. net ME energy (e), net ME energy vs. polar and charged residue's frequency, except 4J15 (green)(f), net ME vs. Hoop-Woods hydrophilicity (g) and net ME energy vs Kyte-Doolittle hydrophobicity (h).

This enzyme functions in mesophilic and extremophilic conditions in the cytoplasm. Although it can be seen from RMSD (Table 1 - see PDF) that their topologies are almost similar in reference to human enzyme, the primary sequences of extremophiles are 50-60% different from each other and from that of the mesophile (Figure 1b). It is also visible on the phylogenetic tree (Figure 1a). It should be noted here that our dataset consists of mesophilic (4J15) and extremophile representatives. Extremophilic includes halophilic (1HM5) and thermophilic representatives. Interestingly, thermophilic includes Euryarchaeota (1B8A), Crenarchaeota (1WYD) and thermophilic bacteria (1N9W). The main purpose of the present work is to know the fundamental contribution of salt-bridges in making the topology the similar, despite the fact that the sequences are quite different (see below). Comparing the frequencies of two types of salt bridges, namely ip and nu, with that of the mesophilic reference (human) does not make much difference (data not shown). Although Euryarchaeota and Crenarchaeota have more salt-bridges of ip type than mesophile (human), nu type has no such difference. Interestingly, on the other hand, if we compare the total energy of ip and nu or their sum with that of human, considerable differences are visible (Table 1 - see PDF and Figure 1c). Not only that, the total ME energy of ip and nu type salt-bridges or their sum is similarly higher than that of human (Table 1 - see PDF and Figure 1d). Again, the net-energy obtained from the sum of the three component energy terms is highly correlated with ME energy (Figure 1e). It means the ME is determinant of net-energy. It needs to be mentioned here since the desolvation and the bridge terms negate the effect of each other and the absolute value of their sum is less, ME, compared to the salt-bridge, can be of charged, polar, hydrophobic in nature. Again, a positively or negatively charged ME can orient itself in a favorable, unfavorable, or neutral manner against the two oppositely charged partners of the salt-bridge. Therefore, in the case of ME energy, these three possibilities are equally likely. As a matter of fact, regardless of the nature of the salt-bridge and the domain of life, we see that net ME is always favorable (Figure 1). Here, it is pertinent to ask, how ME relates to the properties of the sequence. To resolve this question, we have examined ME relations with various amino acid classes and their grand average properties. We have divided ME-residues into four types namely ab (charge), po (polar), pg (Pro and Gly) and hb (hydrophobic). Since ab frequency is comparatively more (also higher in extremophiles than human) than other classes, we expected ab's contribution to ME-energy to be greater. Conversely, at ME's energy contribution point of view, po has overtaken ab. In fact, ab class is unfavorable in humans and thermobacteria, which extremophiles have surpassed. It seems that at least in the present study, when ab class makes ip and nu salt-bridges, po class enriches the ME. These results are presented in Figure 1f-h. In this context, few points are noteworthy. First, among all classes, sum of ab and po classes has shown strong correlation (r2 0.96) with ME energy except for the human (Figure 1f). Second, Hoop-Wood's grand average hydrophilicity, which relies on the amino acid residues, is inextricably correlated (r2 = 0.95) with net ME energy (Figure 1g). Kyte-Doolittle grand average hydrophobicity, on the other hand, is in complete disarray (r2 = 0.37) from such a relationship (Figure 1h). Fourth, the structures, 1HM5 has been obtained through the homology model. Surprisingly, it restores the salt-bridges like the X-ray structures and places the ME residues properly and favorably (Figure 1). Overall, ME is particularly concerned with the properties of the sequence, which means that ME will be able to gain access to the intrinsic codes of the sequence.

**Figure 2:**
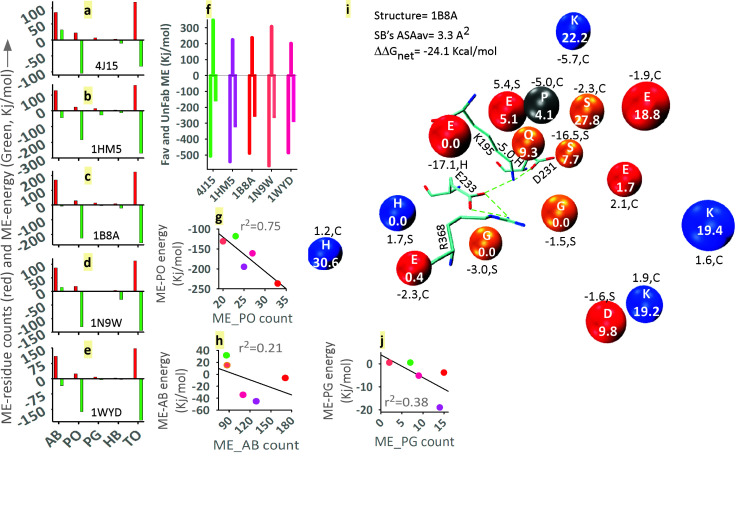
Microenvironment and its favorable and unfavorable energy and energy contributions in relation to residue-class. a-e: Charge (ab), polar (po), pg, hydrophobic (hb) and to (total) class specific ME energy contribution; f: Protein specific unfavorable (upper positive bars), favorable (much-lower negative bars) and net ME energy (small-lower negative bars); g-j: Correlation of ME energy and residue class with respect to proteins; i: Description of the components and features of ME. The letter written on the beads indicates ME-residue, whose color is their nature (acidic, basic and polar). The numbers on the beads are their accessibility. The side number (positive or negative) of the beads indicates the ME-residue's interaction energy and the letter (H, helix; S, strand; C, coil) indicates the secondary structure.

We have shown that both mesophilic and extremophilic cases have favorable ME energy. Now, we have presented Figure 2 to understand the basis of this. Conceptually, a protein is a thermodynamically compromise state [1, 36]. Therefore, it is normal to have favorable and unfavorable forces in it. In fact, in the case of each protein, we see an unfavorable and favorable force within ME (Figure 2f). Then, how is the favorable force more in extremophiles? The favorable force is almost the same for all these proteins (∼-500 Kj/mol) but the unfavorable force is more in human (∼350 Kj/mol). This means that extremophiles have gained more stability by reducing the unfavorable ME components than human. In the case of thermobacteria, unfavorable ME-component is more than archaea. At the same time, the favorable component in this case has also been alleviated (Figure 2f).

### ME properties and its favorable and unfavorable population:

Looking at the amino acid classes, it is clear that among the four classes (ab, po, pg and hb), there is an additional contribution to the favorable energy of the po class (Figure 2a-e). Notably, the frequency (red-bar, Figure 2a-e) of ab (acid and base) is much higher than that of po (polar), but in terms of energy (green-bar, Figure 2a-e) contribution, it is po. Although ab is more, its energy is unfavorable in the case of human and thermobacteria (Figure 2a,d), which has surpassed the extremophiles (Figure 1b, c and e). Significantly, the contributions of pg and hb are negligible in all respects (Figure 2). It is understood now that in the correlation of frequency of (ab+po) vs. ME-energy (Figure 1f), polar (po) residue's contribution is major. This incident has also been revealed in the separate correlation plots of po vs. ME-energy (Figure 2g) and ab vs. ME-energy (Figure 2h). A typical ME has been described to explain the composition and binary details of the microenvironment (Figure 2i). Although the salt-bridge is in the core (i.e. ΔΔGdslv is very high), the net-stability is -24.1 kcal/mol. We have taken this representative salt-bridge from 1B8A. In general, we know that ab and po are less probable at the core of protein, since there is less chance of neutralization of these isolated charges and dipoles [6]. In contrast, here, the ME is made up of base (blue), acid (red) and po (orange) residues (Figure 1i). Half of these residues are in coil and the rest are present in helix and strand. The most interesting thing is that, overall, MEs make a favorable contribution to the net-stability of the salt-bridge. Such salt-bridges are rarely seen in normal mesophilic proteins [37]. There is also controversy over whether the core's salt-bridge is stable. We need to emphasize here that the number of stable network salt-bridges (50-60%) in our dataset is higher in the core. It is important to remember that we compute nu's net-energy by NUM [14].

### Mutagenesis of ME-residue:

**Figure 3:**
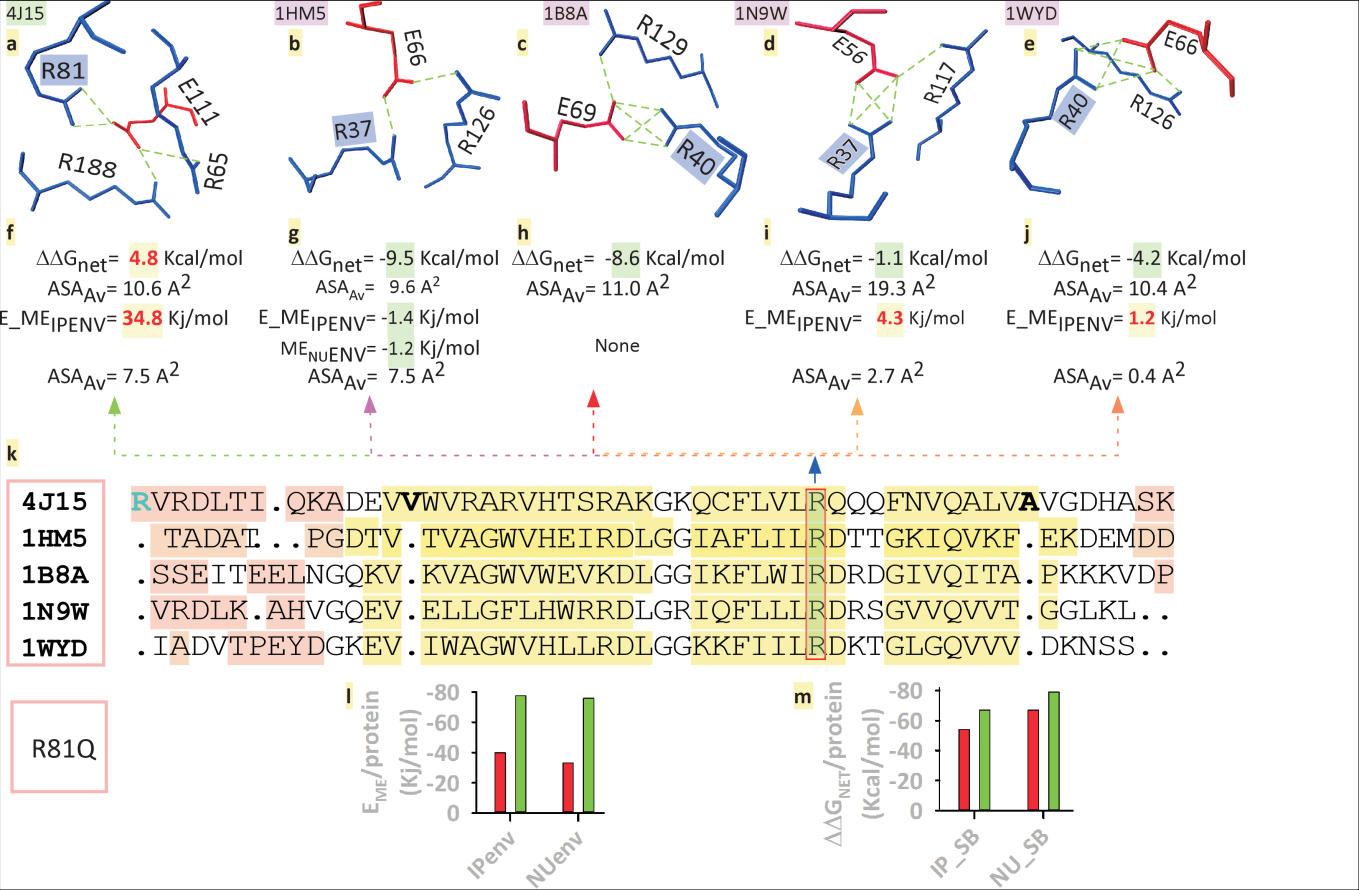
Conserved and unfavorable ME-residue's in silico mutation and its effects. a-e: The conserved residue, Arg (in blue shade) participates in the network salt-bridge in each protein; f-j: The energy of the salt-bridge and participating MEs (ip and or nu); l-m: Change in stability in MEs (ipenv and nuenv) and salt-bridges (ip and nu) of the mutant protein (green) compare to the wild-type (red).

There are important applications in the practical and fundamental research of protein engineering [39]. Proteins are characterized by site-directed mutagenesis and molecular dynamic simulations analysis [39- 41]. However, it is not known how protein's ME affects its structure, stability, and function. Highlighted residue (R) is conserved in all proteins is in the sheet (Figure 3k). The residue is used as a candidate in network salt-bridge (Figure 3a-e) and ME residue in every protein (Figure 3f-j). Nevertheless, in the case of 4J15, this residue makes both network and ME unstable (Figure 3f). Although the network has surpassed it, this instability is somewhat visible in the case of ME (Figure 3i and j) in thermobacteria and Crenarchaeota. In this respect, halophilic and Euryarchaeota have exceeded the instability (Figure 3g-h). In the case of human, we mutate that residue from R to Q (i.e. R81Q) through homology modeling method. The total energies of the salt-bridge (ip_sb and nu_sb) and the ME (ipenv and nuenv) are, then, compared between the wild and mutant proteins (Figure 3l-m). In the case of mutant protein in ME's, both ipenv and nuenv have more than doubled the stability of the wild type protein (Figure 3l). Similarly, mutant protein, for R81Q, has gained ∼= -10 Kcal/mol additional energy in networked and isolated salt-bridges. Since R81Q affects the energy of both the salt-bridge and the ME, it can have a global effect.

## Discussion:

The protein sequence contains the codes of structure and function, which can only be decoded in a real environment. [2]. Just as proteins incorporate evolutionary pressure at the same time, they can also maintain their topology for biological function. This probably helps extremophiles to live in a deliberate style in the extreme (high-temperature, high-salt, etc.) environment [3,4,10, 29,31,41]. The phylogenetic distances of thermophiles and halophiles compared to humans seem to be proportional to the difference in the homologous position of their sequences. Not only is that, but also the constraints of the environments in their sequences also reflected in their different phylogenetic positions. In order to understand the effect of these differences on the weak force, we compare and analyze the salt-bridge and its ME. Salt-bridge's high net-stability in extremophilic enzymes seems to be due to its bypassing environmental stress [4]. The high correlation between ME-energy and net-energy, residue-frequency, and hydrophilicity suggests that MEs play a vital role in protein's stability. This is even more so because, out of the three component terms, the costly desolvation and the contributing bridge energies neutralize each other. Thus, the ME appears to be the major determinant of the net-stability of the salt-bridge [14-16]. Here, mention may be made of the fact that the distance, orientation, geometry of the ME-residues plays a major role, subject to the opposite charges of the salt-bridge [12,29]. Deviation from these criteria is, therefore, a source of unfavorable energy that is always associated with protein folding. Nevertheless, the surprise is that in all cases, the favorable ME energy is always more than the unfavorable energy. Again, in the case of extremophiles, while the favorable energy is almost comparable as that of the human, the unfavorable energy is much less. Overall, it can be said that the evolution of the extremophiles has replaced the human's unfavorable ME-residues with the favorable ones.

Isolated-charges and dipoles can be the main cause of instability in protein folding [14,6]. If they are at the core, salt-bridge can stabilize them. It should be noted here, that in the case of extremophiles, the number of stable core salt-bridges is higher than that of the surface. Interestingly, we have seen that as these salt-bridges increase, so do the isolated ab and the po residues. Since ME is a major determinant of net-stability of salt-bridge, more costly desolvation energy of core salt-bridge can only be overcome if ME has more. To improve ME, it seems that extremophilic sequences are more hydrophilic [10,11] than human and for this reason, the isolated ab, and po residues at the core are higher. Thus, it is obvious that although the salt-bridge is in core and even though desolvation cost is high, it is stable under the influence of ME. Whether the core salt-bridge is stable or not is an area of active debate today. In most cases, more desolvation costs of core salt-bridges have been reported [30]. Nevertheless, ME seems to be working impeccably. Overall, the increase in hydrophilicity of sequence, which is a case in extremophilic proteins, is to enrich ME, to stabilize salt-bridge.

Protein engineering can be used to solve many unresolved questions in relation to protein's structure and function [43,44]. Many lethal mutations alter the function of proteins and causing serious diseases. Gene therapy can overcome these problems [43]. From the study here, we can understand which residue needs to be changed. The energy and binary details of ME appears to be especially useful in protein engineering. The target residue, although conserved, is unfavorable to human and thermobacteria, but stable in extreme proteins. The residue is important because it is participating as a ME residue as well as a partner of the salt-bridge. The extremophiles have left the target residue intact, removed one partner from the nu, and recruited ME to go from unfavorable to favorable. This means that in the case of human, we can stabilize the protein by altering the target residue. Our method analyzes the protein structures and extracts the ME residues from which the unfavorable, favorable, and binary details can be obtained using crystal as well as homology-modeled structures [21]. Proteins of unknown structures can also be analyzed using the latter method. We think that this information from ME will work in genetic engineering and molecular dynamic simulation. Overall, the ME of proteins seems to be of paramount importance in extracting and understanding the problematic or useful residue from the structure to the sequence of protein.

## Conclusion

The presence of isolated charges (ab) and polar (po) residues in the core of extremophilic proteins helps in the formation of stable salt-bridges with reduced desolvation. Thus, site-specific mutations with favorable residues at the ME will help to develop thermo stable proteins with strong salt bridges.
